# Technology-Dependent Rehabilitation Involving Action Observation and Movement Imagery for Adults with Stroke: Can It Work? Feasibility of Self-Led Therapy for Upper Limb Rehabilitation after Stroke

**DOI:** 10.1155/2022/8185893

**Published:** 2022-10-29

**Authors:** Sheree A. McCormick, Christopher Ireland, Abebaw M. Yohannes, Paul S. Holmes

**Affiliations:** ^1^Department of Psychology, Manchester Metropolitan University, Manchester, UK; ^2^Graphical Data Ltd., Northern Ireland, UK; ^3^Department of Physical Therapy, Azusa Pacific University, California, USA

## Abstract

**Background:**

Motor (re)learning via technology-dependent therapy has the potential to complement traditional therapies available to older adults living with stroke after hospital discharge and increase therapy dose. To date, little is known about the feasibility of technology-dependent therapy in a home setting for this population.

**Objective:**

To develop a technology-dependent therapy that provides mental and physical training for older adults with stroke and assess feasibility. Specifically we ask, “Can it work”?

**Design:**

Single group repeated measures.

**Methods:**

13 participants, aged 18 years and over, were recruited over a six-month period. All participants had mild upper limb impairment following a stoke and were no longer receiving intensive rehabilitation. All participants received 18 days of technology-dependent therapy in their own home. Information was gathered on recruitment and retention, usability, and suitability of outcome measures.

**Results:**

11 participants completed the study. The recruitment rate (number recruited/number canvassed; 10.7%) suggests 1907 participants would need to be canvassed to recruit the necessary sample size (*n* = 204) for a definitive trial designed to provide 90% power at 5% level of significance to detect a clinically meaningful difference of 5.7 points on the Action Research Arm Test. The usability of the application was rated as exceptional on the System Usability Scale. Effectiveness cannot be determined from this study; however, there was a trend for improvement in measures of upper limb function and emotional well-being. *Limitations*. The study was limited by a relatively small sample size and lack of control group.

**Conclusions:**

This study demonstrated proof of concept of a technology-dependent therapy for upper limb rehabilitation following stroke. The data suggest a definitive trial is feasible, additional strategies to improve recruitment should be considered. Outcome measures aligned with the residual motor function of participants are required.

## 1. Introduction

Stroke remains one of the leading causes of death and disability worldwide [1]. Reports suggest 70% of individuals who have had a stroke experience upper limb or arm weakness with approximately 62% not regaining useful upper limb function at six months [2]. Positive engagement in high intensity and task specific rehabilitation has been recommended for physical and emotional recovery after stroke [3, 4]; however, issues such as cost and access often limit the dose of rehabilitation provided following hospital discharge. The unprecedented new challenges to patient care determined by the COVID-19 pandemic, for example, physical distancing and avoidance through fear of catching disease, have further constrained therapy provision for community dwellers with stroke. Mobile tablet-based therapies for stroke rehabilitation have the potential to address many of these challenges, however, when developing these technologies, there is a need to ensure new barriers to therapy engagement (e.g., system, device, and usability) are not introduced [5, 6].

Another novel approach that may improve therapeutic treatment and patient outcome is movement (re)learning via mental simulation techniques [7, 8]. These techniques typically include action observation (AO), the process of observing and understanding an action in the absence of sensory input [9], and mental imagery (MI) defined as process that enables us to represent perceptual information in our minds in the absence of actual sensory input [10]. Jeannerod's Simulation Theory [11] suggests that the AO, MI, and physical execution of goal-directed movements activates common neural areas of the motor system. As such, the motor pathways associated with either process (AO, MI, or physical execution) may be enhanced via any of the other two through Hebbian learning. [12]. In support of this assumption, AO and MI have been reported to improve motor performance in music [13], sport [14], aviation [15], surgical practice [16], and movement rehabilitation [9, 17].

Traditionally, evidenced-based rehabilitation programs targeting upper limb impairment for stroke focus on occupational and physical therapy modalities. Increasingly, novel “top-down” treatments such as AO and MI are being considered adjuncts to these therapies [7, 8, 18–20]. When used in conjunction with physical therapies, there is evidence that these techniques can increase functional cortical regeneration in damaged motor areas through neuroplasticity, defined as “the ability of the nervous system to change its activity in response to intrinsic or extrinsic stimuli by reorganizing its structure, functions, or connections” [9, 21–23]. Reported as having no known adverse effects [24], the inclusion of these movement (re)learning techniques in self-led outpatient rehabilitation programs may improve patient outcomes without the costs typically associated with clinician-delivered physical therapies.

A convenient and effective solution proposed to overcome accessibility barriers in therapy provision is telerehabilitation; provision of clinician-led therapy guided remotely via information communications technology. Despite growing popularity, a recent Cochrane report found limited evidence of low or moderate quality to support its use and no studies that examined cost-effectiveness [25]. The concept is appealing as a method of improving reach and dose, however, new barriers relating to digital technology may be inadvertently introduced; delivery requires a broadband connection, an application to run the video, technology support, a device capable of handling the technology, and an agreed appointment time between therapist and patient. Some of these critical elements are not available to all, particularly those from disadvantaged groups or residing in undeveloped countries. An alternative, more accessible solution could be personalized, self-led therapy using a stand-alone technical device [5, 6, 26].

Using of a stand-alone technical device to deliver rehabilitation has been employed in MusicGlove therapy [27], Virtual Glove therapy [28], ArmeoSenso [29] (virtual reality system), iTSA [30] (smart tablet to treat post-anomia), Rehabilitation Gaming System (RGS; virtual reality system) [31], and observation therapy [32, 33]. Some, but not all, of these studies used “light touch” support from a therapist, and all reported preliminary evidences that self-led physical and/or observational therapy can improve outcomes following stroke.

Despite its appeal as a relatively low-cost and effective adjunct to physical therapy in stroke, few studies [17, 22, 26, 32, 34, 35] have included explicit mental movement (re)learning techniques in home-based, self-led therapy. Early work by Holmes and Ewan [17] reported a case-study of a novel at-home personalized AO and physical practice intervention to aid stroke rehabilitation, demonstrating that systematic observation of personalized and meaningful videos over a 12-week period can support functional improvement and emotional well-being. The findings were corroborated in a larger randomized control trial by Dettmers and colleagues [32] who demonstrated that a six-week intervention of home-based video training was highly acceptable, did not cause any side-effects, and improved hand function, activities of daily living (ADLs) and quality of life. In a pilot study Dijkerman and colleagues [22] reported that four weeks of self-led, home-based movement imagery improved performance on the trained task but improvements were not generalized to other ADLs. More recently, Fuchshofer and colleagues [26] demonstrated “above average” usability ratings of the prototype ANIMATE app, a digital intervention to stimulate the motor system in subacute stroke through observing and imagining six different ADLs. Collectively, these studies suggest movement (re)learning techniques may provide additional opportunities for people living with stroke to perform valid rehabilitation activities independent of direct clinical intervention and based in their own home. We are not aware of any studies in stroke that have combined contingent mental (AO and MI) and physical movement (re)learning techniques in a home-based, self-led application. We propose that personalized technology-dependent therapy (TDT), used in this way, could enhance early supported discharge and help to meet the recommendations for enduring task-specific rehabilitation [3, 36].

This study reports the development of the *See, Imagine, Move: Upper Limb Action Therapy* (SIMULATe) iPad™-based application and the results of an early feasibility study. Data relating to recruitment, retention, adherence, patient experiences, and suitability of outcome measures were gathered to establish whether a definitive randomized control trial (RCT) is warranted [37].

## 2. Method

The study was approved by the UK National Health Service Research Ethics Committee and was conducted in accordance with the Declaration of Helsinki. Registration of the proof-of-concept study in a trial database was deemed nonmandatory by the UK National Health Service Research Ethics Committee. All participants provided written informed consent. Following recommendations [38], this report is aligned with the TIDieR (Template for Intervention Description and Replication; see supplementary file (available here)) Checklist [39].

The aim was to develop a personalized TDT combining the physical execution, AO and MI of goal-directed movements to support upper limb rehabilitation following stroke. The objectives were to (i) develop the application using principles of user-centred design (UCD), ISO 9241-210 [40], and (ii) assess the feasibility of the TDT for a future RCT: could sufficient participants be recruited and retained, was the application usable, were outcome measures appropriate, and could they be collected in a valid and reliable manner?

### 2.1. Development of the Application

UCD is an approach based on the needs and interests of the user, with an emphasis on making products usable and understandable [41]. When a UCD is used long-term, engagement with the system (i.e., the degree of voluntary use of a system over a period of time) may be improved [42]. UCD employs the following cocreation principles (see Gulliksen et al. [43]): (i) the design is based on an explicit understanding of users, tasks, and environments; (ii) users are involved throughout design and development; (iii) the design is driven and refined by a user-centred evaluation; (iv) the process is iterative; (v) the design addresses the whole user experience; and (vi) the design team includes multidisciplinary skills and perspectives.

The research team comprised a usability designer, a cognitive-behavioral psychologist, a physiotherapist, and a software development company. Three public and patient involvement and engagement (PPIE) representatives, a Stroke Association UK Coordinator, and an occupational therapist were consulted in three steering group meetings during the project. An early prototype of SIMULATe was demonstrated to seventeen physical and occupational therapists from two hospitals in the North-West of the UK; clinicians provided feedback on the functional design and user interface.

### 2.2. Specification for Context of Use and User Requirements

The initial design criteria for SIMULATe were drawn from evidenced-based practitioner guidelines: the revised applied model of deliberate imagery [44], the delivery factors for AO [45], and the modified PETTLEP model of MI [46]. The application's design and functionality were initially storyboarded by the usability designer using low-level prototyping tools, paper and pencil, whiteboard, and Microsoft PowerPoint™. This low-cost approach reduced the need for software to be developed prematurely, permitted the development team to get a “look and feel” for the application, and allowed the concept of the tool to be discussed in a meaningful way with stakeholders [43].

### 2.3. Evaluate against Design Requirements

The application was evaluated by the PPIE representatives and clinicians during the development phase. The usability designer was present at all design meetings to ensure consistency. The purpose of the evaluation sessions was to further determine users' needs and usability goals. This information was used to build a profile of a virtual user to inform the design process. A virtual profile represents distinct groupings of meaningful behaviors, goals, and motivations of the target user. Clinicians and individuals with stroke were consulted as it was anticipated that their views could differ; clinicians evaluating recovery using defined quantitative bench marks and individuals with stroke focusing more on activities that brought meaning to their life pre-stroke [47].

A prominent profile characteristic identified through discussions in the early design phase was independent learning. The need to design an application with large, intuitive icons, moderate error tolerance, and minimal set-up and navigation requirements was highlighted. The PPIE representatives suggested that the application included an instructional video with commentary and help pages and each training element (AO, MI and physical practice) to be preceded by written prompts to guide the user through the therapy session. These multimodal features, which support individual learning styles [48] promoting acceptability [49], were built into the functionality.

In the initial design and with a strong focus on established motor cognition mechanisms, SIMULATe included discrete actions known to activate the putative human mirror neuron system (reach, grasp, and pinching type actions). Feedback from the steering group and clinicians revealed the need for the therapy to be constructive, engaging, and person-centred. To address these needs, the reach, grasp, and pinch actions were embedded within typical ADLs. The ADLs were grouped into categories reported to be important in stroke rehabilitation: dressing (doing up a zip), entertainment (games such as cards or dominoes), eating and drinking, household chores (such as polishing, folding laundry), and personal care [50]. This approach was aimed at promoting engagement by allowing users to select actions to meet their personal and functional needs. Engagement was also encouraged by providing feedback to the user, through the application's graphical user interface, regarding the number of minutes of therapy performed each day.

The steering group suggested that task complexity be matched to the functional ability of the user. To achieve this, functionality was developed to allow each action to be observed either as a complete sequence or deconstructed into segments (i.e., component parts). For example, the action of “putting toothpaste on a toothbrush” involved reaching for a tube of toothpaste, grasping it, twisting the top off, grasping the toothbrush, squeezing or pinching the toothpaste tube, directing the toothpaste onto the toothbrush, and raising the toothbrush to the mouth in a coordinated manner. One component part of this action is unscrewing the top off the toothpaste tube. Simulating actions as either a sequence or as a component part follows occupational therapy guidelines that suggest complex tasks should be deconstructed into component parts with the eventual aim of reconstructing the action as a whole sequence when the patient has acquired sufficient skill [51]. SIMULATe was designed to offer the user the opportunity of selecting similar component parts (e.g., a two-point pinch) from different action contexts (e.g., pinching a pen and pinching a sweet). This functionality allowed the user to repeatedly practice the component part mentally and physically but in a variable context. This approach has been suggested to support better performance, retention, and transfer [51].

### 2.4. Final Design

**Figure 1:**
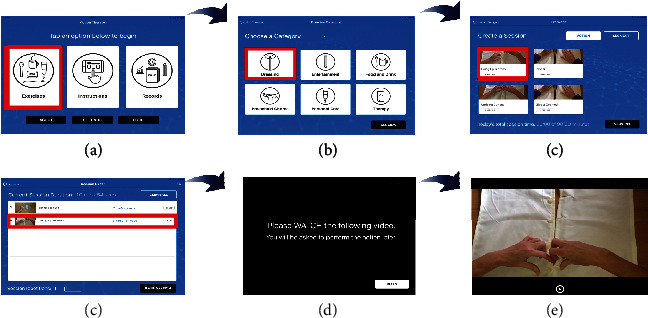
The schematic illustrates how an exercise session is created and executed. In a typical session a user would (a) select the exercise programme; (b) select an ADL category; (c) select an action(s) from within the category, using the top right-hand buttons to toggle between *action* or *segment*; (d) add the action(s) to the exercise session; (e) start the session; and (f) begin SIMULATe training (in this example, doing up buttons from a first-person visual perspective is presented). Note: prior to selecting the exercise program, the dominant hand is selected in the default settings.

**Figure 2:**
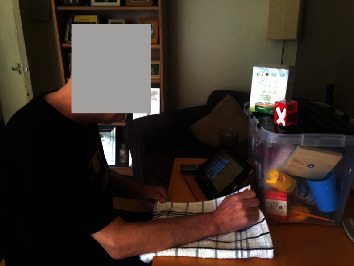
An individual using the SIMULATe application at home to practice one of the ADLs in the library (pinching the ends of a tea towel and folding it over). The individual in this manuscript has given written informed consent to publish this photograph.

A flow diagram of SIMULATe's functionality is shown in Figure 1, and its use in an applied setting is shown in Figure 2.

For each ADL selected, the application guides the user through two-three minutes of action observation, 45 seconds of motor imagery, and two minutes of physical practice. Once completed, the application progresses to the next ADL in the exercise session or returns to the main menu.

The ADLs in the AO videos were presented from first- and third-person visual perspectives to support contingent kinesthesis and spatial awareness of the person-task interaction [12]. The upper torso of a model (either a male or female, 45 years of age) sat at a table in an upright chair was filmed. All actions were performed without upper-limb impairment to assist the user in seeing and imagining a competent, best possible “self” movement [52]. It is possible that observing an action in the third person and imagining in a first person perspective could limit the dependency on visual coding [53], although this concept requires testing.

## 3. Feasibility Testing

Thirteen community dwelling individuals with stroke were recruited through community stroke clubs, the Stroke Association UK and social media. Participants were included in the study if they (i) were between three and 60 months poststroke (ischaemic or hemorrhagic), (ii) were able to raise their most affected upper limb from their lap to the table top (from a seated position), (iii) could follow a two-stage instruction to observe and imitate a single action with their least affected upper limb, (iv) were over 18 years of age, and (v) had the mental capacity to provide written informed consent. Patients were excluded if they were able to complete the nine hole peg test (9-HPT) with their most affected limb in under 30 seconds.

### 3.1. Design

**Figure 3:**
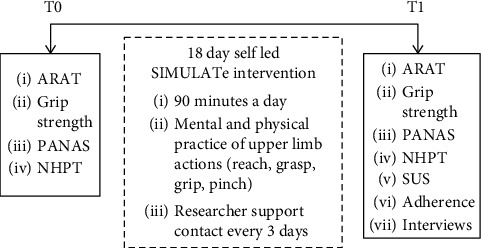
A schematic of the study design. Outcome measures include the action research arm test (ARAT) [54, 55], grip strength, positive and negative assessment scale (PANAS) [56], nine hole peg test (9-HPT), System Usability Scale (SUS) [57], and adherence and semistructured interviews.

The study was initially planned as a mixed method between groups design (1 × control group, 1 × intervention group). Recruitment was unexpectedly slow, and ethical approval was obtained to change the design to a mixed method within-group design to provide sufficient proof-of-concept data and meet project deadlines (Figure 3). Outcome measures were assessed at T0 and T1; between T0 and T1, the SIMULATe intervention was applied.

### 3.2. Procedure

At the initial home visit, each participant was provided with the SIMULATe application downloaded onto a tablet computer (iPad™) and a box of task related accessories, for example, toothbrush, toothpaste, cup, screw top jar, knife, and fork playing cards, required to perform each ADL. Based on previous research [9] a daily target therapy time of 90 minutes (1620 minutes over 18 days) was prescribed. Participants were informed that the therapy was self-led and that the amount of therapy they performed each day would be recorded on the tablet. During the initial visit, following written informed consent and baseline assessments, a SIMULATe training session was held with each participant (and primary carer if required). At the end of the training session the participant was asked to use SIMULATe to practice an ADL of their choice. This task was observed to ensure the treatment was enacted as designed.

Specific exercise sessions were not prescribed during the intervention, participants were asked to select personally meaningful actions from a library of 45 upper limb ADLs. Participants were contacted every three days by their preferred method (telephone, or email) to answer questions and ensure they were not experiencing any difficulties.

### 3.3. Outcome Measures

Primary outcome measures included recruitment and retention rates, usability, and adverse effects. The recruitment rate was calculated as the number of people recruited as a percentage of the number of people invited to participate. The retention rate was calculated as the number of completers as a percentage of the number of people recruited. Usability was assessed using the System Usability Scale (SUS) [57]. The ten-item scale provides a global assessment of the subjective experience in terms of its effectiveness, efficiency, and satisfaction. Responses were scored on a five-point Likert scale ranging from 1 (Strongly Disagree) to 5 (Strongly Agree). Scores were translated to 0–100%, with a higher score representing better usability. Scores above 90 are considered exceptional, above 70 promising, and below 50 generally considered to have usability issues that are cause for concern [58, 59]. Adherence (frequency of use recorded by the SIMULATe software) and patient experiences (captured through semi-structured interviews at T0) are described in a separate report.

The following secondary measures (pre- and posttest) were administered.

#### 3.3.1. Action Research Arm Test (ARAT)

The ARAT [55] is a 19-item measure divided into 4 subtests (grasp, grip, pinch, and gross arm movement). Performance on each item is rated on a 4-point scale ranging from the following: 3—performs test normally; 2—completes test but takes abnormally long or has great difficulty; 1—performs test partially; and 0—can perform no part of test (scores range from 0-57, floor and ceiling effects, respectively). The standardized scoring protocol published by Yozbatiran et al. [55] was employed.

#### 3.3.2. 9-HPT

The standardized set-up and execution procedure outlined by Mathiowetz et al. [60] was employed.

#### 3.3.3. Grip Strength

Grip strength was measured using the Jamar dynamometer (model 2a, hydraulic, analog, anatomical grip, and 5 positions) and the standardized method described by Roberts et al. [61]. Maximum grip strength was recorded as the mean of three attempts, the force production dial was hidden from view, and verbal encouragement was given.

#### 3.3.4. Positive and Negative Affect Scale (PANAS)

The PANAS [56] is a 20-item questionnaire measuring emotional well-being. The assessment includes 10 items for the positive measure and 10 items for the negative measure. Scores on the positive and negative scale range from 10 to 50, floor and ceiling effects, respectively. Participants are asked to rate the extent to which they have experienced pleasurable engagement (including emotions such as enthusiasm and alertness) and unpleasurable engagement (characterized by subjective distress) in the past week on a 5-point Likert scale.

### 3.4. Data Analysis

As this study assessed feasibility and not treatment effectiveness, descriptive statistics describe the outcome measures. For pre- and posttest measures, the median and interquartile range (IQR), are reported.

### 3.5. Role of the Funding Source

The project was funded by NIHR Brain Injury HTC Innovations Small Funding Competition and a Manchester Metropolitan University Knowledge Exchange and Innovative Fund Award. The funding was used to meet all consumable and PPIE costs and software development of the SIMULATe application.

### 3.6. Results of Feasibility Testing

#### 3.6.1. Primary Outcome Measures

Eleven participants (mean [±SD] 56.3 ± 9.8 years; 20.3 ± 15.7 months poststroke; 7 males) completed the study. Ten participants were right-handed, and for 6 participants, their dominant side was most affected by the stroke. In accordance with the ARAT classification used by others [62], participants had mild loss of arm function at baseline; ARAT 43.0 (39.5-57.0). The characteristics of those recruited to the study are shown in Table 1.

#### 3.6.2. Recruitment, Retention and Adverse Effects

**Figure 4:**
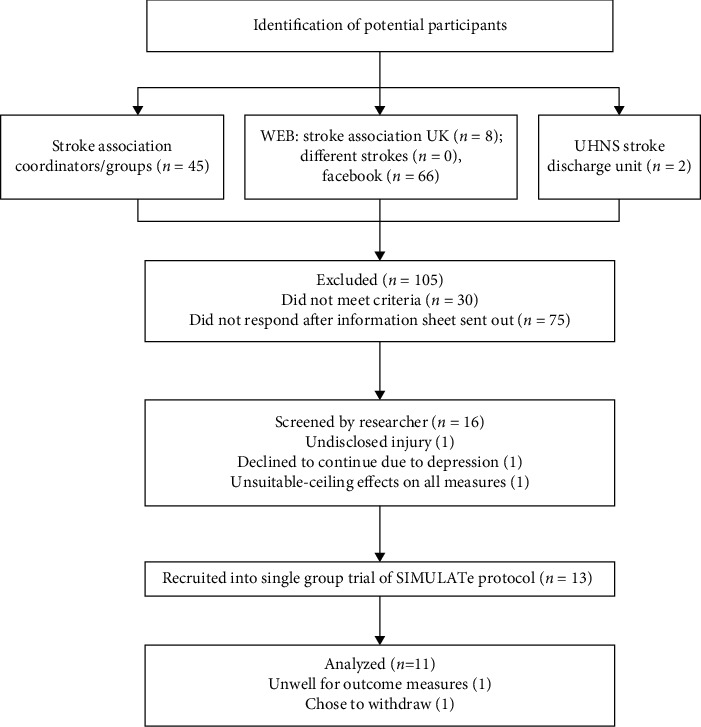
CONSORT diagram illustrating participant recruitment and retention.

Participants were recruited over a 6-month period through community-based services and social media (https://www.facebook.com/groups/differentstrokesuk; https://www.stroke.org.uk). The CONSORT flowchart [63] (Figure 4) illustrates the screening, recruitment, and retention throughout the study. 121 individuals were identified as potential participants, 30 were excluded following initial email/telephone prescreening, and 75 did not respond to the participant information sheet. Of the remaining 16, 3 did not meet the inclusion criteria. Thirteen participants provided written informed consent resulting in a recruitment rate of 10.7%. The retention rate was 84.5%; one participant withdrew due to a serious adverse event unrelated to the study, and one participant withdrew voluntarily commenting that she preferred to do other things with friends.

#### 3.6.3. Usability

The SIMULATe application scored 91.2 (87.5-92.5) on the SUS.

#### 3.6.4. Secondary Measures (Reported as Median and IQR)

**Figure 5:**
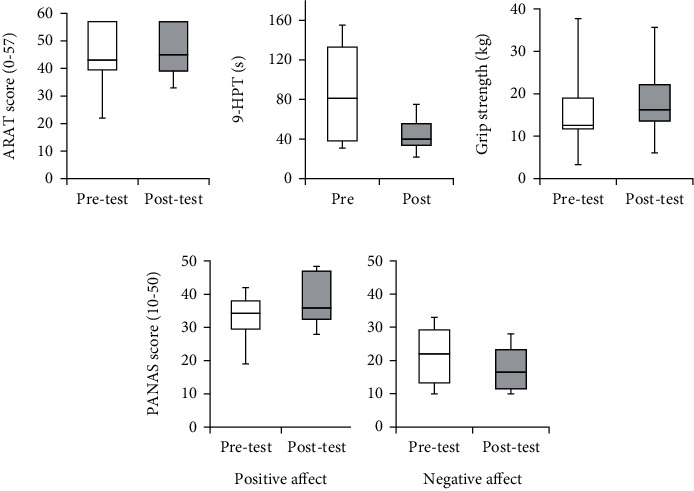
Box plots of the secondary outcome measures; (a) ARAT [55], (b) 9-HPT, (c) grip strength, and (d) PANAS [56]. Horizontal lines indicate the median values, boxes indicate the interquartile range, and vertical lines indicate the full range.

All secondary outcome measures showed a trend for improvement. The data is presented to illustrate change in each measure for the purpose of outcome selection for a future trial. The ARAT improved from 43.0 (39.5-57.0) to 45.0 (39.0-57.0); ceiling effects were measured in three participants at baseline (Figure 5(a)). Improvements were observed in the 9-HPT (*N* = 7∗) with movement time decreasing from 81.1 s (38.2-132.8) to 39.9 s (33.5-55.7) (Figure 5(b)). Grip strength improved from 12.8 kg (11.8–20.1) to 16.1 kg (13.7–22.2) (Figure 5(c)). The PANAS (*N* = 10∗) indicated an improvement in affect; positive affect increased from 34.2 (29.5–38.0) to 36.0 (32.5–47), and negative affect decreased from 22.0 (13.2–29.2) to 16.5 (11.5–23.3) (Figure 5(d)). ^∗^Four participants were unable to complete the 9-HPT within the recommended time limit (300 s) due to an inability to pinch all the pegs; one participant elected not to complete the PANAS for personal reasons.

## 4. Discussion

This study has demonstrated early proof of concept of TDT for upper limb rehabilitation following stroke. The findings suggest a definitive RCT is feasible and warranted, with some modifications to recruitment strategies and outcome assessments.

### 4.1. Recruitment and Retention

Recruitment to this study presented some challenges. It took six months to recruit 13 participants despite using a variety of recruitment methods—community teams, a stroke discharge unit, and social media. Using the standard deviation from the ARAT in this trial (11.35) and the retention rate (84.6%), the estimated sample size to provide 90% statistical power (*α* = .05, 2-tailed test) for a multicentre RCT of SIMULATe is 102 per group to detect a clinically meaningful 5.7 unit change on the ARAT. Using this sample size as a guide and assuming three centres nationwide were involved, it could take up to 32 months to recruit the required sample and an estimated 636 people would need to be canvassed at each centre. These data suggest a fully powered RCT would require additional strategies to support recruitment in a timely manner.

Recruitment strategies in this study included professional referral from the Stroke Association UK coordinators, consultant referral from a stroke discharge unit at the University Hospital North Staffordshire, UK and self-referral from social media sites and stroke groups. Recruitment from one stroke group accounted for 23% of the sample. Recruitment was poor from the other three stroke groups (7%). Recruitment from the Stroke Association UK coordinators in Crewe, UK, accounted for 31%. Some coordinators from other areas reported that they did not have the resources to identify eligible participants. There were two referrals from the stroke discharge unit but neither progressed to recruitment. The most successful recruitment strategy was via social media (https://www.facebook.com/groups/differentstrokesuk; 38%). These data suggest that traditional methods of recruitment may not be the most practical way to reach potential participants living in the community and that alternative methods (e.g., social media) may be more efficient and effective.

Difficulties in recruiting individuals with stroke to home-based studies have been reported by others [28]. Standen et al. [28] assessed the feasibility of a home-based rehabilitation program using a low-cost virtual reality system. Their study adopted broad inclusion criteria and worked with community teams and a stroke unit. A low recruitment rate (similar to this study) was reported; 27 participants randomized over a 15-month period. Our data indicate the most effective source of recruitment was an established on-line stroke community suggesting people living with stroke have, and use, web-based technology at home. We did not have ethical approval to collect reasons for refusal to participate; however, it is possible that patients who declined may have had little knowledge about emerging and adjunctive therapies such as mental imagery and action observation. To address this, a future trial may wish to offer action observation and mental imagery taster sessions to potential participants considering trial participation.

### 4.2. Usability

SIMULATe's high ratings of usability across participants, two of which had not used an electronic tablet before, suggest that the application has good to excellent acceptance in the field and supports earlier reports that stand-alone applications can be used to facilitate rehabilitation in individuals who have had a stroke [26, 64].

### 4.3. Suitability of Outcome Measures

This study was not powered to assess effectiveness; however, median values for all secondary outcome measures showed a trend for improvement. This is promising given that participants were in the chronic stage of stroke when rehabilitation outcomes are often less favorable. Ceiling effects on the ARAT were recorded for three participants at baseline, and floor effects were observed on the 9-PHT in four participants (participants could not pick up the pegs due to a lack of preservation in finger extension). These findings highlight the difficulties associated with assessing motor function across the broad spectrum of post-stroke upper-limb dysfunction. To overcome these challenges, Thompson-Butel et al. [65] recommend selecting assessments that are aligned with the residual motor function of participants. In accordance with Thompson-Butal et al. [66], a subsequent RCT could retain the ARAT and 9-HPT to measure performance in participants with moderate-low and high motor function, respectively. In addition, the Box and Block Test (a test in which the participant transports 2.5 cm^3^ wooden cubes from one compartment of a box to another) could be used to measure performance in participants with moderate motor function.

Similar to the findings of others who have measured psychosocial well-being during stroke rehabilitation [67], median scores on the PANAS showed a trend for improvement in emotional well-being; scores for positive affect increased and scores for negative effect decreased pre-post trial. Although speculative, improvements in emotional well-being could be linked to an increase in self-efficacy, perhaps through mastery or vicarious experiences (physical, imagined, or observed). Future research may wish to include a comprehensive battery to better understand what is driving change in this domain and explore if affect can be enhanced further using targeted AO and MI techniques in this population.

### 4.4. Cost-Effectiveness

This study did not assess clinical cost-effectiveness. The high degree of usability and acceptability of the SIMULATe application, however, suggests it has the potential to provide low-cost unsupervised home training which could be economically viable in comparison with conventional rehabilitation. Furthermore, OFCOM's Adults' Use and Media Report 2018 [68] indicated that more than a quarter of older adults (75+) are using electronic tablets, an increase of 15% since 2015. These data suggest tablets are becoming increasingly common in the homes of older adults potentially reducing costs associated with TDT provision. A cost-effectiveness analysis in comparison to conventional therapy will be required in future SIMULATe studies in the home setting.

## 5. Limitations

The study was limited in terms of the small sample of moderate-high functioning participants. The study may have been influenced by bias as the researcher conducting assessments was not blind to the study. That said, primary outcomes in this study included recruitment and retention which are relatively independent of bias. Future studies should address these issues by including a larger sample, blinded assessors, and fidelity checks to ensure training is delivered and received as per protocol.

## 6. Conclusion

In summary, the findings from this study suggest highly usable TDT can be designed and developed with people living with stroke, for people living with stroke. A future RCT is feasible and warranted. We recommend using additional and innovative strategies to boost recruitment and stratifying participants by upper limb motor function, using assessments that are aligned with the residual motor function of the participant. Enhancing specific invention components to further improve emotional well-being should be explored.

## Figures and Tables

**Table 1:** Baseline characteristics of participants who completed the study (*n* = 11).

	*N* (%)
Gender	7 (63.6)
Male	
Dominance	10 (90.9)
Right	
Side of hemiparesis	6 (54.5)
Right	
	Mean (SD); range
Age (years)	56.3 (9.8); 40-71
Months post stroke	20.3 (15.7); 6-46
ARAT	44.4 (11.4); 22.00-57.00
Grip strength (kg)	34.9 (22.2); 7.33-85.67
Nine hole peg test (s)	96.3 (51.6); 31.0-155.0
PANAS	Positive affect 32.9 (7.3); 19-42 Negative affect 21.2 (9.0); 10-33

## Data Availability

The anonymised data used to support the findings of this study are available from the corresponding author upon request.
